# Berlin Letter

**Published:** 1913-12

**Authors:** Charles Haase

**Affiliations:** Luisen Platz II


### CORRESPONDENCE.

---

*This department is intended for the presentation of news and views not coming within the scope of other departments, and particularly to afford opportunity for discussion and criticism of views elsewhere expressed.*

---

### Berlin Letter

Berlin, October 26, 1913.

There are now about sixty American physicians working in the clinics here. The following from our neighborhood:

Dr. Henry Buswell, Buffalo; Dr. A. A. Mitten, Buffalo; Dr. Douglas Arnold, Buffalo; Dr. E. C. Koenig, North Tonawanda, N. Y.; Dr. L. B. Amsbry, Utica, N. Y.; Dr. C. Haase, Elmira, N. Y.

At a meeting of the Anglo-American Medical Association, held on October 25, 1913, the following officers were elected:

President, Dr. F. B. Mowbray, Hamilton, Canada.

Vice-President, Dr. Charles Haase, Elmira, N. Y.

Secretary, Dr. Douglas Arnold, Buffalo, N. Y.

Treasurer, J. J. Reilly, St. Louis, Mo.

Prof. Körte gave a lecture on "Surgical Treatment of Pancreatic Tumors and Inflammations."

On October 27, 1913, Prof. Dr. Schleich, in Prof. Kraus's clinic, showed about forty cases that had been treated with Friedmann's "tuberculosis serum." They were cases of bone and gland tuberculosis. Many of the cases of bone tuberculosis were demonstrated with X-ray plates taken at various stages of the treatment. The cases had improved or were free from all signs and symptoms. In many of the cases various other methods had been tried with no improvement. These patients had been treated by Dr. Schleich. Prof. Kraus (and there is no better medical man in Germany) has been using F.'s germs for the past two months. He has given about 200 injections. He did not care to express definite opinion, but did say that it had done no harm to any of his cases and that he was inclined to look favorably upon it.

Dr. Friedmann was called upon to talk, and he said "he wished the medical profession would forget the past and carefully try his method out. They would be glad to teach the method to any physician free of charge.

Am pleased to see the BUFFALO MEDICAL JOURNAL at the A. A. M. A. Club Rooms.

Best wishes,
CHARLES HAASE,
Luisen Platz II.

